# Postoperative In-Hospital Morbidity and Mortality of Patients With COVID-19 Infection Compared With Patients Without COVID-19 Infection

**DOI:** 10.1001/jamanetworkopen.2021.5697

**Published:** 2021-04-12

**Authors:** Max R. Haffner, Hai V. Le, Augustine M. Saiz, Gloria Han, Jeffrey Fine, Philip Wolinsky, Eric O. Klineberg

**Affiliations:** 1 Department of Orthopaedic Surgery, University of California, Davis, Sacramento; 2 Division of Biostatistics, University of California, Davis, Davis

## Abstract

This cohort study uses the Vizient Clinical Data Base to compare the postoperative in-hospital morbidity and mortality of surgical patients with COVID-19 infection with patients without COVID-19 infection.

## Introduction

Several small studies have suggested that patients with positive test results for COVID-19 infection may experience worse perioperative outcomes and increased mortality after surgery.^1,2,3,4,5,6^ However, those studies were underpowered and lacked generalizability and comparative cohorts.^1,2,3,4,5,6^ This study used data from a national database to compare the clinical outcomes of surgical patients testing positive for COVID-19 infection with those of a matched sample of surgical patients testing negative for COVID-19 infection. Elucidating the comparative surgical risk profiles of patients with and without COVID-19 infection would help health care systems to improve preoperative guidelines and clinicians to better inform patients in shared medical decision making before surgery.

## Methods

This was a retrospective cohort study of hospital discharge data from the Vizient Clinical Data Base (Vizient Inc). Patients 18 years or older with and without COVID-19 infection who underwent surgery from April 1 through November 30, 2020, were matched on a 1:1 ratio based on age and case mix index. Procedures among all surgical specialties were included. Patients who underwent gynecologic, obstetrical, or minor procedures (eg, tracheostomy, percutaneous cardiovascular procedures) were excluded. Inpatient mortality, complications listed in the Vizient Clinical Data Base, patient safety indicators, hospital-acquired conditions, and length of stay were compared between the cohorts. Subgroup analyses were performed to compare outcomes among public, private, and nonprofit hospital systems. The University of California, Davis Institutional Review Board approved the study and waived patient informed consent because the data were deidentified. This study followed the Strengthening the Reporting of Observational Studies in Epidemiology (STROBE) reporting guideline.

The χ^2^ or Fisher exact test was used to evaluate the association between COVID-19 status and, if death occurred, the presence of complications listed in the Vizient Clinical Data Base and patient safety indicators. The Mann-Whitney test was performed to evaluate whether a difference in length of stay based on COVID-19 status existed. All statistical analyses were performed with SAS version 9.4 (SAS Institute Inc). Hypothesis tests were 2-sided and evaluated at a significance level of *P *< .05.

## Results

A total of 5470 surgical patients with positive COVID-19 test results were matched with 5470 surgical patients with negative COVID-19 test results during the same study period. Among all hospitals, there were more than double the number of deaths reported in the cohort of patients with COVID-19 (811 [14.8%]) compared with the cohort of patients without COVID-19 (388 [7.1%]) (*P* < .001). The rates of complications listed in the Vizient Clinical Data Base (818 [15.0%] vs 760 [13.9%]; *P* = .11) and median length of stay (10.0 [interquartile range (IQR), 1.3-36.4] vs 10.7 [IQR, 1.0-558.0] days; *P* = .86) did not differ significantly between the 2 groups. However, hospital-acquired conditions (110 [2.0%] vs 46 [0.8%]; *P* < .001) and patient safety indicators (183 [3.3%] vs 129 [2.4%]; *P* = .002) were higher in patients with COVID-19 (Table 1).

**Table 1.** Patient Characteristics, Frequencies, and χ^2^ Test *P* Values by COVID-19 Status

Characteristic	COVID-19 status, No. (%)		*P* value^a^
	Negative (n = 5470)	Positive (n = 5470)	
Patient death			
Yes	388 (7.1)	811 (14.8)	<.001
No	5082 (92.9)	4659 (85.2)	
Complications			
Yes	760 (13.9)	818 (15.0)	.11
No	4710 (86.1)	4652 (85.0)	
Hospital-acquired conditions			
Yes	46 (0.8)	110 (2.0)	<.001
No	5424 (99.2)	5360 (98.0)	
Patient safety indicators			
Yes	129 (2.4)	183 (3.3)	.002
No	5341 (97.6)	5287 (96.7)	
Length of stay, median (IQR), d	10.7 (1.0-558.0)	10.0 (1.3-36.4)	.86

Abbreviation: IQR, interquartile range.

^a^ Mann-Whitney test *P* value for surgical patients by COVID-19 status.

Within each hospital ownership type (public, private, nonprofit), more deaths occurred in the group with COVID-19 compared with the group without COVID-19 in public hospitals (146 [15.8%] vs 46 [4.8%]; *P* < .001) and nonprofit hospitals (631 [14.7%] vs 326 [7.5%]; *P* < .001), but not in private hospitals (34 [14.1%] vs 16 [9.4%]; *P* = .15). Among surgical patients with COVID-19, there were no differences in mortality rates, complications listed in the Vizient Clinical Data Base, hospital-acquired conditions, or patient safety indicators among public, private, or nonprofit hospitals (Table 2).

**Table 2.** Patient Characteristics, Frequencies, and χ^2^ Test *P* values by COVID-19 Status and Hospital Type

Characteristic	Hospital type									*P* value^b^
	Public (n = 1890)			Private (n = 412)			Nonprofit (n = 8638)			
	COVID-19 status, No. (%)		*P* value^a^	COVID-19 status, No. (%)		*P* value^a^	COVID-19 status, No. (%)		*P* value^a^	
	Negative (n = 968)	Positive (n = 922)		Negative (n = 171)	Positive (n = 241		Negative (n = 4331	Positive(n = 4307		
Patient death										
Yes	46 (4.8)	146 (15.8)	<.001	16 (9.4)	34 (14.1)	.15	326 (7.5)	631 (14.7)	<.001	.62
No	922 (95.2)	776 (84.2)		155 (90.6)	207 (85.6)		4005 (92.5)	3676 (85.4)		
Complications										
Yes	119 (12.3)	132 (14.3)	.20	24 (14.0)	33 (13.7)	.92	617 (14.2)	653 (15.2)	.23	.69
No	849 (87.7)	790 (85.7)		147 (86.0)	208 (86.3)		3714 (85.8)	3654 (84.8)		
HACs										
Yes	10 (1.0)	16 (1.7)	.19	2 (1.2)	6 (2.5)	.34	34 (0.8)	88 (2.0)	<.001	.72
No	958 (99.0)	906 (98.3)		169 (98.8)	235 (97.5)		4297 (99.2)	4219 (98.0)		
PSIs										
Yes	34 (3.5)	24 (2.6)	.25	5 (2.9)	9 (3.7)	.65	90 (2.1)	150 (3.5)	<.001	.38
No	934 (96.4)	898 (97.4)		166 (97.1)	232 (96.3)		4241 (97.9)	4157 (96.5)		
Length of stay, median (IQR), d	11.7 (2.2-29.4)	9.5 (1.0-95.0)	<.001	12.8 (3.7-22.7)	9.0 (1.0-64.0)	<.001	10.2 (1.3-36.4)	11.0 (1.0-558.0)	<.001	<.001

Abbreviations: HACs, hospital-acquired conditions; IQR, interquartile range; PSIs, patient safety indicators.

^a^ Mann-Whitney test *P* value for surgical patients by COVID-19 status and hospital type.

^b^ Compares only surgical patients with COVID-19 by hospital type.

## Discussion

In this retrospective cohort study of 10 940 surgical patients, the findings suggest that COVID-19 infection positivity was an independent risk factor for increased perioperative mortality but not complications. Specifically, the overall mortality rate in the cohort with COVID-19 (14.8%) was more than double that in the cohort without COVID-19 (7.1%). To our knowledge, this study represents the largest comparative cohort study between surgical patients testing positive for COVID-19 and those testing negative for the virus, and it is the first to compare outcomes among different hospital settings. This study has 2 main limitations. Patient outcomes could not be compared by clinical severity of COVID-19 infection. Furthermore, we could not determine specific types of surgery or whether the surgery was elective, urgent, or emergent.

Our study findings suggest that COVID-19 infection positivity is an independent risk factor for surgical mortality. In addition to using scarce medical resources and placing health care workers at risk of exposure, surgery for patients with COVID-19 poses a safety risk given the increased mortality rate observed in this study. As the COVID-19 pandemic continues and surges, we need to balance patients’ surgical needs with COVID-19–specific risks in the setting of a strained health care system. Surgical patients with COVID-19 should be informed of their higher in-hospital mortality risk. More important, postponing surgery should be recommended for patients with a positive preoperative COVID-19 test result when possible unless surgical intervention is absolutely necessary for life- or limb-saving measures.
